# Fenestrated and Branched Stent-Grafts for the Treatment of Thoracoabdominal Aortic Aneurysms: A Systematic Review and Meta-Analysis

**DOI:** 10.3389/fcvm.2022.901193

**Published:** 2022-05-31

**Authors:** Zhongzhou Hu, Zheng Zhang, Hui Liu, Zhong Chen

**Affiliations:** Department of Vascular Surgery, The Capital Medical University Affiliated Beijing Anzhen Hospital, Beijing Institute of Heart Lung and Blood Vessel Diseases, Beijing, China

**Keywords:** thoracoabdominal aortic aneurysm, endovascular repair, fenestrated and branched stent-graft, systematic review, meta-analysis

## Abstract

**Purpose:**

To investigate the safety and efficacy of total endovascular repair for thoracoabdominal aortic aneurysms (TAAAs) with fenestrated and branched stent-grafts.

**Methods:**

The MEDLINE, EMBASE, and Cochrane databases were searched between January 2001 and December 2021 to identify literature relevant to the use of fenestrated and branched endografts for the treatment of TAAAs. Studies with <4 cases and those on juxtarenal or pararenal aortic aneurysms were excluded. Meta-analyses were conducted to evaluate spinal cord ischemia (SCI), irreversible SCI, renal insufficiency, dialysis, endoleak, reintervention, target vessel patency, 30-day mortality and overall mortality. Fourteen studies comprising 1,114 patients (mean age 72.42 years, 847 men) were selected. The mean TAAA diameter was 67 mm. The Crawford TAAA classification was type I-III in 759 cases, type IV in 344 cases, and type V in 10 cases. Outcomes of the meta-analysis are reported as proportions and 95% confidence intervals (CIs).

**Results:**

The pooled rates for 30-day mortality and overall mortality were 6% and 18%, respectively. The pooled rate for technical success was 94% (95% CI, 93–96%), for SCI was 8% (95% CI, 7–10%), for irreversible SCI was 6% (95% CI, 4–7%), for reversible SCI was 5% (95% CI, 4–6%), for reversible SCI was 2% (95% CI, 2–3%), for renal insufficiency was 7% (95% CI, 5–10%), for dialysis was 3% (95% CI, 2–4%), for target vessel patency was 98% (95% CI, 97–99%), and for reintervention was 15% (95% CI, 9–24%).

**Conclusion:**

Fenestrated and branched endografts for the treatment of TAAAs are safe and effective with acceptable early results. Lifelong regular follow-up and additional prospective studies are necessary to substantiate whether this technique is valid.

## Introduction

Untreated large-diameter thoracoabdominal aortic aneurysms (TAAAs) have a total rupture rate of more than 26% (1). However, the treatment of TAAAs is greatly challenging for vascular surgeons. Since Etheredge et al. (2) first successfully completed an open surgery for a patient with a TAAA in 1955, surgical repair has become the gold standard of treatment.

Even so, due to the substantial trauma that results from the extensive incision and the durable blocking of blood flow to the visceral arteries, conventional open surgery is still associated with notable mortality and morbidity (2, 3). The evidence concerning the usage of β-blockers and angiotensin receptor blockers (ARBs) to decrease aneurysm expansion remains subject of debate (4). To date, no class of drug has been shown to be effective. Reduction of cardiovascular risk are recommended, such as antiplatelet, lipid-lowering and antihypertensive therapy (5). If the TAAA diameter exceeds certain dimensions, timely preventive surgical or endovascular intervention is necessary. Since 2001, Chuter et al. (6) has successfully accomplished total endovascular repair of a TAAA with branched stent-grafts, and total endovascular repair with fenestrated and branched stent-grafts has constantly evolved and become a minimally invasive alternative option for the treatment of TAAAs, especially for patients who may not tolerate open surgery. Although increasing reports on this endovascular technique have indicated encouraging outcomes (7–9), there have been few meta-analyses regarding total endovascular TAAA repair using fenestrated and branched stent-grafts to date.

The purpose of this study was to systematically review the literature on fenestrated and branched stent-grafts for total endovascular TAAA repair and to perform a meta-analysis to evaluate the safety and efficacy of this endovascular technique.

## Methods

### Literature Search

Two investigators independently performed a thorough search of the MEDLINE, EMBASE, and the Cochrane databases from January 2001 through December 2021 to identify English-language articles eligible for this study. After searching, the literature was checked by two independent researchers blinded to study design. The following combination of keywords was used for all databases: “fenestrated endograft,” “multibranched,” “branched,” “endograft,” “graft,” “stent,” “stentgraft,” “stent-graft,” “t-brach,” and "thoracoabdominal aortic aneurysm (TAAA).”

The studies were selected according to the following inclusion criteria: those (1) with high-risk patients with a preoperative diagnosis of TAAAs, (2) with patients who experienced complete endovascular repair using either fenestrated or branched endografts or both, (3) with >3 cases, and (4) that clearly described patient demographics, as well as primary technical success rates, mortality rates, stent patency, clinical outcomes of complications and follow-up times. The exclusion criteria were as follows: studies (1) with <4 cases, (2) reporting on juxtarenal aortic aneurysms and/or pararenal aortic aneurysms, and (3) focused on open surgery or hybrid methods.

### Data Extraction

Data were extracted regarding the demographics of patients (mean age, number of patients, sex and preoperative comorbidities); morphology of the TAAAs (Crawford TAAA classification and mean aneurysm size); procedure (technical success rate, number of target vessels, number and location of lost target vessels, main endograft model, models of branch stents, mean procedure time, mean volume of contrast and mean fluoroscopy time); and outcome (target vessel patency, 30-day mortality, overall mortality, reasons for death, type and number of reinterventions, stent migration, complications, and mean length of follow-up).

### Study Selection

**Figure 1:**
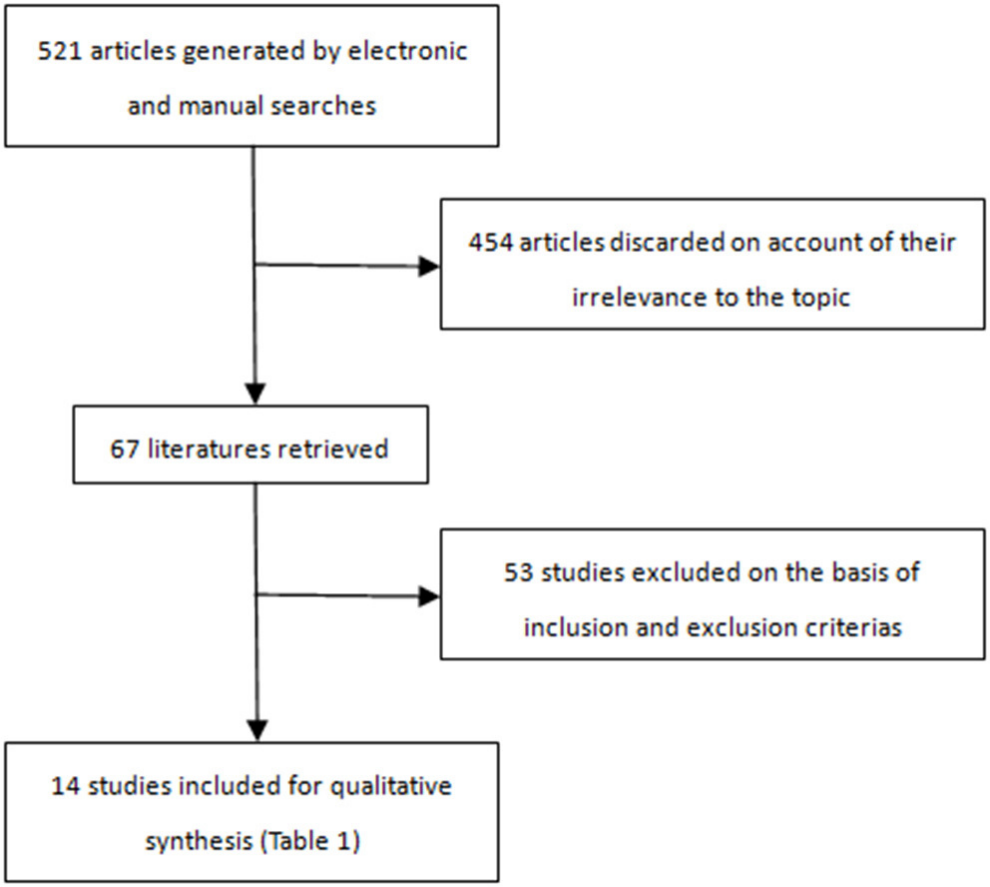
Study flowchart.

A total of 521 articles were retrieved and screened (Figure 1); 14 articles (7–20), which included 1,114 patients (mean age 72.42 years; 847 men), were in accordance with the inclusion criteria (Table 1).

**Table 1:** Demographic characteristics.

**Name**	**Study period**	**Patients**	**Follow up (month)**	**Age (y)**	**Sex (m/f)**	**Crawford classification**	**Aneurysm size (mm)**
Anderson et al. (10)	2003	4	12	74	3/1	1I, 2III, 1IV	65
Roselli et al. (11)	2004–2006	73	12	75	56/17	28I-III, 45IV	71
Smith et al. (12)	2004–2007	6	17	71	4/2	2II, 1III, 3IV	70
Haulon et al. (13)	2006–2009	33	33	70	30/3	1I,7II, 12III, 13IV	64
Clough et al. (14)	2008–2011	31	12	71	21/10	12I,13III, 6IV	64
Ferreira et al. (15)	2006–2012	48	8	68	33/15	8I,5II, 5III, 29IV	NA
Harrison et al. (16)	2009–2011	10	NA	73.8	6/4	10II	69
Bisdas et al. (17)	2010–2013	46	9.7	70.5	36/10	2I,13II, 21III, 10IV	59
Iafrancesco et al. (18)	2007–2012	62	23	72	55/7	26I-III, 36IV	NA
Kinstner et al. (19)	2012–2012	8	18	65.5	6/2	2I,3III, 3IV	61.1
Verhoeven et al. (7)	2004–2013	166	29.2	68.8	125/41	12I, 50II, 53III, 41IV,10V	71
Eagleton et al. (20)	2004–2013	354	36	73.5	270/84	128II, 226III	NA
Oderich et al. (8)	2007–2016	185	22	75	134/51	73I-III, 112IV	66
Gallitto et al. (9)	2010–2018	88	36	73	68/20	43I-III, 45IV	65
	**Total**	1,114	26.91	72.42	847/267	759I-III, 344IV, 10V	67

*NA, not available; m, male; f, female*.

These studies were carried out from April 2003 to April 2018 and were published from October 2005 to November 2019. The Crawford TAAA classification was type I-III in 759 cases, type IV in 344, and type V in 10; there was 1 Stanford type B dissection associated with a large TAAA (15). The mean aneurysm diameter was 67 mm. The preoperative comorbidities are summarized in Table 2. In total, 296 patients had a history of previous surgery.

**Table 2:** Comorbidity data.

**Name**	**Hypertension**	**COPD**	**CRI**	**CAD**	**Diabetes**	**Smoke**	**CVD**	**PS**
Anderson et al. (10)	1	2	2	1	0	NA	0	3
Roselli et al. (11)	49	34	19	44	13	NA	8	21
Smith et al. (12)	NA	NA	NA	NA	NA	NA	NA	NA
Haulon et al. (13)	19	14	7	15	2	NA	0	10
Clough et al. (14)	25	5	14	10	2	NA	NA	6
Ferreira et al. (15)	23	7	3	21	10	18	1	17
Harrison et al. (16)	8	0	10	3	0	7	3	2
Bisdas et al. (17)	44	14	NA	38	2	27	0	NA
Iafrancesco et al. (18)	NA	NA	NA	NA	NA	NA	NA	22
Kinstner et al. (19)	8	1	2	2	1	NA	1	NA
Verhoeven et al. (7)	130	97	71	106	13	119	10	78
Eagleton et al. (20)	306	109	75	155	52	306	NA	NA
Oderich et al. (8)	164	88	60	112	31	159	20	87
Gallitto et al. (9)	85	39	38	33	9	NA	10	50
**Total**	862	410	301	540	135	636	63	296

*COPD, chronic obstructive pulmonary disease; CRI, chronic renal insufficiency; CAD, Coronary artery disease; CVD, cerebrovascular disease; PS, previous surgery; NA, not available*.

### Quality Evaluation

All 14 studies were evaluated using Downs and Black template. The median score was 20 (range, 16–22), indicating that the general quality of the included studies was moderate to good.

### Statistical Analysis

This meta-analysis was conducted and presented in terms of systematic reviews and meta-analyses (PRISMA) guidelines (http://www.prisma-statement.org). Separate meta-analyses were conducted for 10 major endpoints: technical success, 30-day mortality, overall mortality, SCI, irreversible SCI, reversible SCI, renal insufficiency (a rise in serum creatinine>0.5 mg/dL), dialysis, reintervention, and target vessel patency. Study heterogeneity was determined using the chi-square-based Q test and quantified using I^2^ statistics. If the I^2^ was <50%, signifying no significant heterogeneity, the meta-analysis was performed using a fixed-effects model; if I^2^ ≥ 50%, the meta-analysis was performed using a random-effects model. The possibility of publication bias was assessed using the Egger test. The results of the meta-analyses are reported as proportions and 95% confidence intervals (CIs). The meta-analyses and the publication bias assessment were performed using R statistical software packages from the R Foundation (http://www.R-project.org).

## Results

### Implanted Stent-Grafts

A total of 1,006 patients were treated using fenestrated and branched endovascular stentgrafts based on the conventional Zenith platform [Cook Medical, Brisbane, Queensland, Australia)], while the Jotec E-xtra design Engineering multibranch stent-graft (Jotec Inc, Hechingen, Germany) was used in 8 patients. The branches of the stent-grafts consisted of 14 stent models: Fluency (Bard Peripheral Vascular, Tempe, AZ, USA); Wallstent (Boston Scientific Corp, Marlborough, MA, USA); Begraft (Bentley InnoMed, Hechingen, Germany); Viabahn (W. L. Gore & Associates, Flagstaff, AZ, USA); S.M.A.R.T. (Cordis Corporation, Bridgewater, NJ, USA); Zilver (Cook Inc); Atrium Advanta (Atrium Maquet Getinge Group, Mijdrecht, the Netherlands); iCast/Advanta V12 (Atrium Medical); Genesis (Cordis Corporation, Bridgewater, NJ, USA); Complete (Medtronic, Minneapolis, MN, USA); Jostent (Abbott Laboratories, Abbott Park, Illinois); Jomed (Abbott Laboratories, Abbott Park, Illinois); Protégé (Medtronic); and Everflex (Medtronic).

### Technical Success and Operative Details

The mean procedure time was 349.4 mins in 748 patients, while the mean duration of radiation exposure was 73.25 mins based on 308 patients. The mean amount of contrast delivered was 173.4 ml in 869 patients. The mean length of hospital stay was 9.51 days in 568 patients. The mean length of stay in the intensive care unit was 48.2 h in 512 patients.

**Figure 2:**
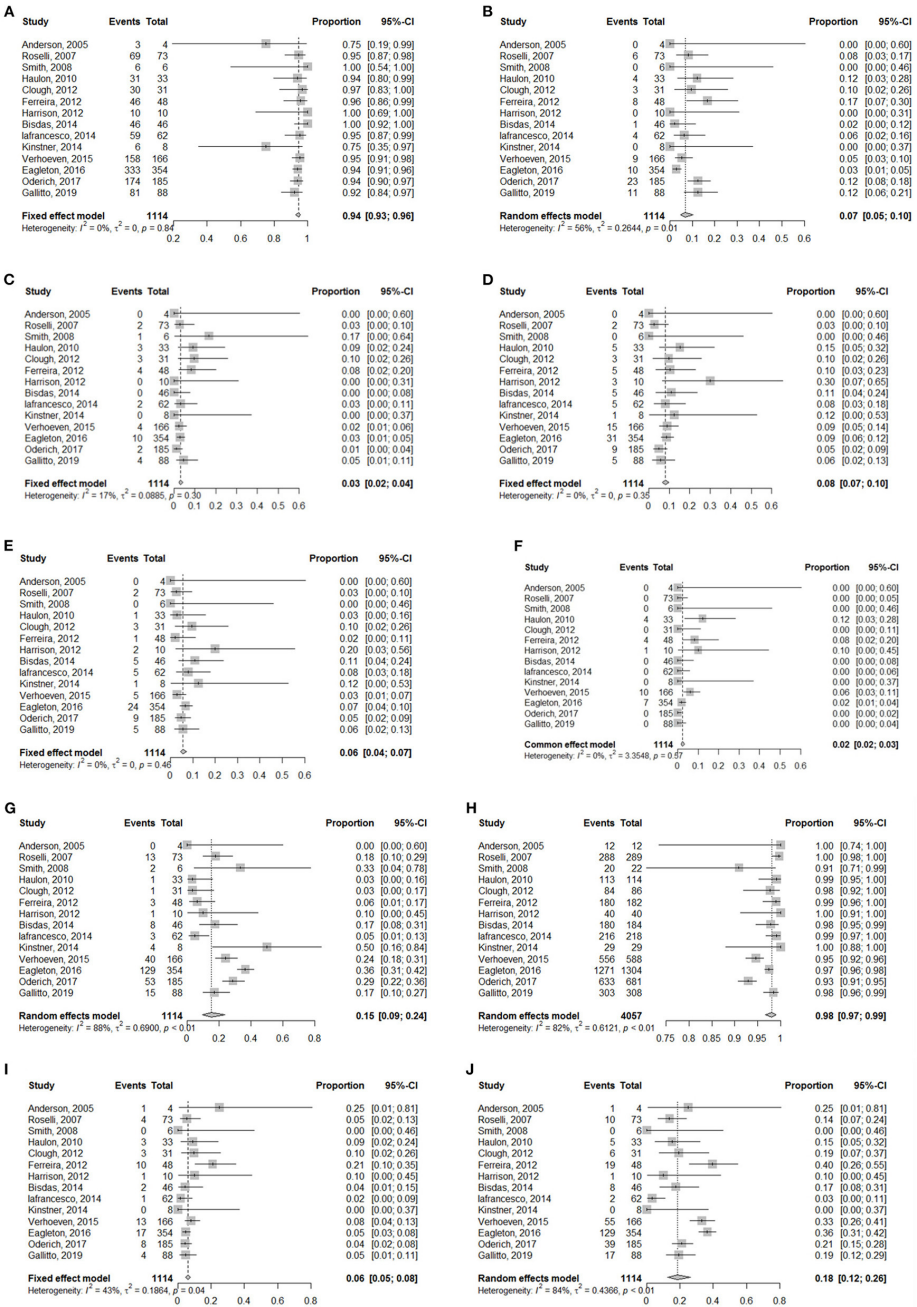
Forest plots for **(A)** Technical success, **(B)** Renal insufficiency, **(C)** Dialysis, **(D)** SCI, **(E)** Irreversible SCI, **(F)** Reversible SCI, **(G)** Reintervention, **(H)** Target vessel patency, **(I)** 30-day mortality, **(J)** Overall mortality.

Technical success (Table 3) was defined as the placement of an aortic stent and all intended target vessels, and absence of angiographic evidence of type I and type III endoleaks was achieved in 1,052 patients. The meta-analysis for technical success showed a pooled proportion of 94% (95% CI, 93–96%) (Figure 2A). On account of high-grade stenosis or occusion or tortuosity of target vessels, aortic kink and maldeployment in aortic grafs. Thirty-two renal arteries in 31 patients and 18 celiac arteries in 18 patients were unsuccessfully incorporated. Among these arteries, 4 renal arteries and 2 celiac arteries were subsequently stented at later times. Five renal arteries in 4 patients were preserved by ilio-renal bypass. One celiac artery was revascularized by a chimney endograft. Renal failure occurred in 2 patients whose renal arteries could not becannulated. One patient refused hemodialysis and died on postoperative day 30 (11). The other patient who had a single renal artery developed acute renal failure combined with a large posterior circulation stroke and died on postoperative day 17 (14). One patient suffered from dissection of all 4 visceral arteries after the placement of bridging stents; revascularization failed, and the patient died (15). One patient experienced incorrect deployment of a distal graft component, which was resolved *via* laparotomy; a renewed attempt to complete the procedure was rejected, and the patient died from MI 4 months after the operation (7). One patient died from intraoperative fatal bleeding resulting from common iliac/vena cava rupture (9). In the remaining 11 patients, the reasons for technical success failure were not explained (8).

**Table 3:** Results after the use of fenestrated and branched stent-grafts for the treatment of TAAAs.

**Name**	**Technical success**	**Target vessels**	**Branch patency**	**RI**	**Paraplegia**	**Paraparesis**	**30-day mortality**	**Overall mortality**	**RV**	**Endoleaks**		
										**I**	**II**	**III**
Anderson et al. (10)	3 (75%)	13	100%	0	0	0	1 (25%)	1 (25%)	0	0	0	0
Roselli et al. (11)	69 (94.5%)	292	99.7%	6	1	1	4 (5.5%)	10 (13.7%)	13	3	7	5
Smith et al. (12)	6 (100%)	22	90.9%	0	0	0	0 (0%)	0 (0%)	2	0	0	2
Haulon et al. (13)	31 (93.9%)	116	99.1%	4	1	4	3 (9.1%)	5 (15.2%)	1	0	4	1
Clough et al. (14)	30 (96.8%)	87	97.7%	3	2	1	3 (9.7%)	6 (19.4%)	1	0	2	1
Ferreira et al. (15)	46 (95.8%)	182	99.5%	8	2	3	10 (21%)	19 (39.6%)	3	0	0	1
Harrison et al. (16)	10 (100%)	40	100%	0	0	3	1 (10%)	1 (10%)	1	0	0	0
Bisdas et al. (17)	46 (100%)	184	97.8%	1	2	3	2 (4.3%)	8 (17.4%)	8	2	0	2
Iafrancesco et al. (18)	59 (95.2%)	221	99.1%	4	0	5	11.6%	23.2%	3	1	0	0
Kinstner et al. (19)	6 (75%)	32	100%	0	1	0	0 (0%)	0 (0%)	4	4	0	2
Verhoeven et al. (7)	158 (95.2%)	593	94.6%	9	2	13	13 (7.8%)	55 (33.1%)	40	14	12	6
Eagleton et al. (20)	333 (94.1%)	1,320	97.5%	18	10	21	17 (4.8%)	129 (36.4%)	129	9	15	43
Oderich et al. (8)	174 (94.1%)	681	93%	23	6	3	8 (4.3%)	39 (21.1%)	53	2	55	29
Gallitto et al. (9)	81 (92%)	317	98.4%	11	3	2	4 (4.5%)	17 (19.3%)	15	1	15	4
Total	1,052	4,100	96.7%	87	32	75	67	315	258	36	110	96

*RV, Reintervention*.

### Morbidity

#### Renal Insufficiency

Renal insufficiency occurred in 87 patients, 35 of whom required dialysis and 9 of whom died. Among these patients needing dialysis, 11 cases were related to renal artery occlusion or stenosis. The meta-analysis for renal insufficiency showed a pooled proportion of 7% (95% CI, 5–10%) (Figure 2B), whereas the meta-analysis for dialysis showed a pooled proportion of 3% (95% CI, 2–4%) (Figure 2C).

#### Spinal Cord Ischemia

The meta-analysis for spinal cord ischemia (SCI) showed a pooled proportion of 8% (95% CI, 7–10%) (Figure 2D). Overall, 54 patients experienced temporary paraparesis, but none had neurological sequelae; 21 patients developed permanent paraparesis. Paraplegia occurred in 32 patients, and 4 cases were persistent. The meta-analysis for irreversible SCI showed a pooled proportion of 6% (95% CI, 4–7%) (Figure 2E). The meta-analysis for reversible SCI showed a pooled proportion of 2% (95% CI, 2–3%) (Figure 2F).

Other morbidities included cardiac complications, pulmonary complications, stroke, subdural hematoma, sepsis, pancreatitis, mesenteric ischemia, ileus, inguinal hematoma, herpes zoster, deep venous thrombosis, subcapsular hematoma of the liver, gallbladder necrosis, upper extremity ischemia, lower extremity ischemia, brachial nerve injury and retrograde dissection.

### Reintervention, and Target Patency

The meta-analysis for reintervention showed a pooled proportion of 13% (95% CI, 7–23%) (Figure 2G). A total of 258 secondary operations were performed. In addition to endoleaks, reintervention was most commonly carried out for branch occlusion/stenosis (n = 57), access complications (n = 25), aortic-related sequelae (n = 13) and lower limb symptoms (n = 8).

A total of 4,100 target vessels were planned to be incorporated. Of these target vessels, 4,053 target vessels were cannulated. The meta-analysis for overall target vessel patency showed a pooled proportion of 98% (95% CI, 97–99%) (Figure 2H). Overall, 96 target vessels were occluded or stenosed during the mean 26.9-month follow-up. With the exception of 54 target vessels without detailed classification (7, 8, 20), 40 target vessels were occluded: 29 renal arteries, 8 celiac arteries and 3 superior mesenteric arteries; stenosis occurred in 2 renal arteries.

### Mortality

The meta-analysis for 30-day mortality showed a pooled proportion of 6% (95% CI, 5–8%) (Figure 2I). Table 4 summarizes the causes of 30-day mortality. The meta-analysis for overall mortality showed a pooled proportion of 18% (95% CI, 12–26%) (Figure 2J). The leading causes of death were aortoesophageal fistula, graft infection, acute dissection of the ascending aorta, pulmonary embolism, myocardial infarction, arrhythmia, cardiac failure, sepsis, renal failure, spinal cord injury, stroke, gastrointestinal bleeding due to multiple organ dysfunction syndrome, and subarachnoid hemorrhage, presumably relevant to a lumbar drain, followed by hydrocephalus and meningitis. The other patients died due to the presence of preexisting comorbidities as a result of reasons related to aneurysms or their treatment.

**Table 4:** Causes of 30-day death.

**Cause of death**	***n***	**%**
Aortic artery rupture	2	3
Iliac/vena cava rupture	1	1.5
Illiac artery rupture	2	3
Cardiac complications	11	16.4
MODS	21	31.3
RF	5	7.5
Mesenteric ischemia	4	6
Respiratory failure	4	6
Pulmonary embolism	2	3
Stroke	7	10.4
SH complications	2	3
HF	1	1.5
Pancreatitis	1	1.5
GI bleeding	2	3
Sepsis	1	1.5
ND	1	1.5
Total	67	100

*SH, subdural hematoma; MODS, multiple organ dysfunction syndrome; RF, renal failure; HF, hepatic failure; GI, gastrointestinal, ND, not determined*.

### Changes of Aneurysmal Diameters

The 5 studies (7, 11–13, 19) including 286 patients clearly showed changes of aneurysmal diameters. The diameters of TAAAs in 276 patients avoided enlargement and decreased more than 5 mm in 188 patients. Aneurysmal diameters in 10 patients increased due to endoleaks.

### Risk of Bias

The 3 of 10 endpoints including technical success, target vessel patency, reintervention were of publication bias.

### Sensitivity Analysis

All studies at each endpoint would be single removed to verify the stability of the results. Severe instabilities containing reversed statistical significance or obvious changes in effect estimates were not be found.

## Discussion

Without technical and methodological improvements and the implementation of ancillary strategies, patients with TAAAs will still face extreme clinical hardship since TAAAs cannot be repaired with acceptable morbidity, mortality and durability. Despite adjunctive techniques, such as cerebrospinal fluid drainage, left heart bypass, selective perfusion of the celiac and superior mesenteric arteries, and refined intraoperative and perioperative treatment, perioperative morbidity and mortality for open surgery remain considerable (21, 22). LeMaire et al. (23) reported that the composite adverse outcome rate of 823 patients with TAAAs undergoing open surgery, defined as operative death, renal failure requiring dialysis, stroke, or paraplegia/paraparesis, remained high at 15.9%. A recent meta-analysis involving 9,963 patients showed that the in-hospital mortality and pulmonary complication rates were 11.26 and 23.0%, respectively (24).

Hybrid procedures comprise open debranching of visceral arteries and subsequent endovascular exclusion of the aneurysm. This repair technique, first reported in 1999, was initially promising for a cohort of patients who could not tolerate open surgery (25). Regardless of the advantages of the avoidance of lower visceral ischemia time, supraceliac aortic clamping and thoracotomy, the traumatic incision and closure of extra-anatomic visceral bypass are not negligible (26–30). In addition, the interval between the two stages increases the possibility of aneurysm rupture (31).

Fenestrated stent-grafts are defined as endografts with holes in the main body that are located next to visceral artery orifices and through which covered or bare metal stents can be placed. Branched stent-grafts consist of caudally or cranially directed or helical branches that are able to incorporate bridging stents to vascularize visceral arteries. The use of fenestrated and branched endografts achieves the total endovascular repair of TAAAs without an open body cavity, clamping of crucial arteries, or visceral ischemia. In addition to the fact that early satisfactory results for the endovascular repair of TAAAs with fenestrated and branched endografts have been published (7–9, 17–20), this procedure is deemed to provide a considerable alternative for the treatment of TAAAs.

The currently accepted complete endovascular repair of TAAAs is more technically demanding and more challenging than traditional endovascular operations; even so, the technical success rate in the current meta-analysis was high (94%), tempering the enthusiasm of vascular surgeons for carrying out the procedure. The excellent technical success rate was presumably attributable to the use of operational teams from large-volume top-level centers, exact patient selection and device design based on 3-D imaging reconstruction techniques. Most cases of technical failure were ascribed to high-grade stenosis, occlusion, tortuosity of target vessels, aortic kinking and maldeployed aortic grafts. To limit the initial contrast dose, 4 renal arteries and 2 celiac arteries were subsequently stented during secondary procedures. If the occluded visceral vessels before repair were excluded from the preoperative plan and if the incorporated target vessels during the secondary procedures were included, the technical success rate would increase. An elaborate preoperative stent-placement plan, an increase in experience with endovascular grafting and further problem-solving skills may improve the technical success rates.

SCI is a vital concern associated with this strategy, as this complication can be detrimental. In the studies examined, 86 patients had SCI, of whom 25 patients finally developed permanent SCI. The pooled SCI rate and the pooled irreversible rate were 8 and 6%, respectively, which were favorably comparable to recent open surgery studies (22, 24, 32). The SCI risk with this endovascular treatment seems to be more attributed to anatomic factors relevant to the morbid morphology than to the physiologic consequences associated with aortic clamping and open operation (9). Extensive aortic coverage, occlusion of spinal collaterals, perioperative hypotension and lower-limb ischemia may increase SCI risk (6). Studies in experimental models and in humans have confirmed the concept of the collateral spinal network (33–37). According to this theory, strategies such as the routine maintenance of the left subclavian artery and bilateral hypogastric artery perfusion, the usual use of cerebrospinal fluid drainage in type I, II, and III TAAAs, the sustainability of normotensive patients during the whole procedure, and the early perfusion of the lower limbs and pelvis were performed, which reduced the incidence of SCI to some extent (7–9, 20). In lieu of the article reported by Reilly and colleagues, in which type Ib endoleak was induced to resolve neurological deficits (37), Harrison et al. intentionally created a temporary endoleak to maintain spinal cord perfusion by a sac perfusion branch attached to a branched endograft (16). Even though this staged approach may lower the occurrence rate and severity of SCI (38), the staging procedure is likely to increase the risk of rupture or death.

Fenestrated and branched stent grafts included custom-made stent grafts and off-the-shelf stent grafts. Currently, T-branch was the most commonly used off-the-shelf stent graft. Generally speaking, for the usage of custom-made stent graft, the ideal patient had minimal atherosclerotic debris within healthy aortic segments, suitable brachial and iliofemoral access, and adequate mesenteric and renal target vessels without early bifurcation or occlusive disease (8). For the use of t-branch, the anatomic inclusion criteria was relatively specific and were as follows: (1) Proximal to aneurysm: Non-aneurysmal thoracic aorta fixation segment proximal to aneurysm with an angle of <90°relative to the long axis of the aneurysm, length of at least 25 mm, and diameter measured outer wall to outer wall no <24 mm and no >30 mm. (2) Visceral vessel anatomy: (a) four indispensable arteries with normal diameters, (b) aortic diameter of >25 mm at the region of the branches, (c) distance between the corresponding arterial orifice and the cuff <50 mm, and the line between the arterial orifice as projected on to the vessel wall and the cuff deviating no more than 45° from the long axis of the aorta. (3) Access: (a) appropriated femoral/iliac access compatible with a 22F (8.5 mm outer diameter) delivery system, and (b) brachial or subclavian access compatible with the delivery profile of a 10F or 12F introducer sheath (3.3 or 4 mm outer diameter) (17, 39).

Renal insufficiency remains another concern of postoperative morbidity in connection with fenestrated and branched endovascular repair for TAAAs. Endovascular TAAA repair prevents cross-clamping and ischemia during renal artery incorporation, preoperative renal dysfunction, and challenging device manipulation impairing renal arteries, and nephrotoxic contrast agents may result in renal insufficiency (40). However, there were acceptable outcomes in this meta-analysis: the pooled renal insufficiency rate was 7%, whereas 3% of patients required dialysis. In total, 35 patients needed dialysis, 11 of whom were associated with renal artery occlusion or stenosis. In the case of increasing the success rate of renal artery revascularization, improving the patency of renal arteries, and lowering the volume of contrast agents, the incidence of renal insufficiency may decrease.

The 30-day and overall mortality rates were 6 and 18%, respectively, in the articles from the meta-analysis, which were better than the corresponding rates of 11.26% and 23.39% reported by Moulakakis et al. (24) in their meta-analysis of 30 studies including 9,963 patients who underwent open repair for TAAAs. When the above results were compared with the meta-analysis of 19 articles involving 507 patients who underwent hybrid treatment for TAAAs (31), a similar finding arose. Notwithstanding, the rate of mortality was relatively high, perhaps as a consequence of patients experiencing TAAA treatment with fenestrated and branched endografts being older and having more severe pre-existing comorbidities that made them unable to tolerate open surgery.

### Limitations

First, as a result of the exclusion of articles in which the results of TAAAs and PRAAs and/or JRAAs were analyzed together, the number of cases in this article was restricted. Second, reports from conferences and non-English-language articles were not included in the study. Third, the follow-up period was slightly short due to this relatively new technique.

## Conclusion

The total endovascular repair of TAAAs with fenestrated and branched stent-grafts in nonsurgical candidates is safe and valid, with early encouraging outcomes. Proper patient selection, ameliorative device designs and developed adjunctive products may improve the outcomes of this technique in the future. Lifelong regular follow-up and additional prospective studies are necessary to substantiate whether this technique is durable.

## Author Contributions

ZH: data collection and synthesis analysis. ZH and ZZ: quality assessment of included literatures. ZC and HL: manuscript reversion. ZH, ZZ, HL, and ZC: final approval of the article. All authors contributed to the article and approved the submitted version.

## Funding

The project was supported by the Capital Health Development Research Project (No. 2018-1-2062).

## Conflict of Interest

The authors declare that the research was conducted in the absence of any commercial or financial relationships that could be construed as a potential conflict of interest.

## Publisher's Note

All claims expressed in this article are solely those of the authors and do not necessarily represent those of their affiliated organizations, or those of the publisher, the editors and the reviewers. Any product that may be evaluated in this article, or claim that may be made by its manufacturer, is not guaranteed or endorsed by the publisher.
